# Chest CT tomography vs. intracavitary electrocardiogram guidance in predicting the length of PICC placement

**DOI:** 10.1186/s12893-022-01604-0

**Published:** 2022-05-19

**Authors:** Huimin Dong, Yuxin Zhu, Xin Zhang, Xinxin Yin, Fude Liu

**Affiliations:** 1 Myasthenia Gravis Center, Shijiazhuang General Hospital, Shijiazhuang, Hebei China; 2 Venous Catheter Clinic, Shijiazhuang General Hospital, No. 365 Jiahua South Street, Yuhua, Shijiazhuang, 05000 Hebei China; 3 Shijiazhuang General Hospital, Shijiazhuang, Hebei China

**Keywords:** Chest CT tomography, Intracavitary electrocardiogram, Guidance, PICC placement, Length, Comparison

## Abstract

**Background:**

To evaluate chest computed tomography (CT) compared to intracavitary electrocardiogram (ECG) in predicting the length of peripherally inserted central catheter (PICC) placement and analyzing the accuracy of the positioning methods.

**Methods:**

This study included a total number of 436 patients who underwent PICC placement. The patients enrolled were randomly divided into two groups: ECG group (n = 218, received IC-ECG) and chest CT group (n = 218, received chest CT). The tip length of the catheter in the superior vena cava, the measured length of the catheter and the actual insertion length of the catheter were observed and recorded in the two groups.

**Results:**

The best catheterization rate of tip positioning and the one-time placement rate of tip positioning in ECG group were significantly higher than that in the chest CT group (all P < 0.05). The comfort level and satisfaction rate in ECG group was significantly higher than that of chest CT group (all P < 0.05).

**Conclusion:**

Accurate catheterization length could be achieved by both chest CT and intracavitary electrocardiogram guidance in the process of predicting PICC placement length. However, IC-ECG guided procedure was more worthy of promotion in clinic.

**Supplementary Information:**

The online version contains supplementary material available at 10.1186/s12893-022-01604-0.

## Background

Peripherally inserted central catheter (PICC) is a widely used peripheral venipuncture catheter in clinical practice for intravenous therapy [1, 2]. Despite that PICC provides convenient and safe venous access for patients undergoing PICC placement, there are limitations. Some patients have suffered from thrombosis and other catheter-related complications. Therefore, focus has been laid in clinical research to reduce the related complications of patients with PICC placement. As the continuous progress of intravenous therapy in recent years, many scholars at home and abroad use various body surface pre-measurement combined with X-ray location methods to define the tip position of the catheter [3]. The intracavitary electrocardiogram localization technology uses the metal guide wire in the catheter as a recording electrode of the electrocardiogram. The tip location of the PICC catheter can reduce the occurrence of ectopic catheter through the predictive changes of P wave. It exerts advantages of real-time guidance, no damage, no pollution, stable effect, timely adjustment [4].

The cardiac CT was usually used to guide left ventricular lead implantation for cardiac resynchronization therapy upgrades [5]. In addition, chest CT scan is capable of observing the course of blood vessels and the changes of soft tissue around blood vessels in the pathway of PICC placement [6]. Therefore, the application of chest CT in PICC placement should be plausible and practical, which might be an alternative for ECG. However, there are limited clinical studies regarding on the comparative study of chest CT and intracavitary electrocardiogram (IC-ECG) in predicting the length of PICC placement. Therefore, comparative study was performed to explore the effect of chest CT vs. intracavitary electrocardiogram in the prediction of PICC placement length, with an attempt to provide an insight and theoretical basis for clinical practice.

## Methods

### Clinical background

There included a total of 436 patients undergoing PICC placement in our hospital from August 2017 to December 2019. The patients enrolled were divided into chest CT group (n = 218) and ECG group (n = 218) via random table method. In ECG group, there were 120 male patients and 98 female patients, aged 22–96 years old, the average age was (60.02 ± 10.21) years. In chest CT group, there were 121males and 97 females, aged 23–97 years old, the average age was (60.62 ± 10.12) years. There was no significant difference in general data between the two groups (P > 0.05, as shown Table 1). Our study was approved by the ethics committee of the Shijiazhuang General Hospital. All patients and their families voluntarily participated in the study and signed the informed consent form, and the formulation of this study program was in line with the relevant requirements of the Helsinki Declaration of the World Medical Association.

**Table 1:** Comparison of general information between the two groups

Group	Gender		Weight (Kg)	Height (cm)	BMI (Kg/m^2^)
	Male	Female			
ECG group	121	97	68.01 ± 12.12	163.96 ± 8.21	23.99 ± 3.21
Chest CT group	120	98	68.09 ± 10.21	163.93 ± 8.11	24.09 ± 3.11
t	0.009		− 0.074	0.038	− 0.330
P	0.923		0.941	0.970	0.742

### Selection criteria

*Inclusion criteria*: patients with PICC placement; normal blood coagulation function; normal heart function; normal ECG P wave; no heart conduction block; normal upper limb movement function; no skin damage at the puncture site; informed consent and signed informed consent forms were gained. Exclusion criteria were poor compliance, mental disorders, patients with heart diseases such as atrial fibrillation, P wave abnormalities, catheterization contraindications.

### Methods

The attending physician ordered PICC placement after the evaluation of the blood vessels of the patients and preparation of the necessary items. All patients used the single-lumen three-way valvular PICC catheter produced by Bard Company of the United States, with a length of 60 cm and a specification of 4Fr made of silicone. The process was confirmed twice.

**Fig. 1:**
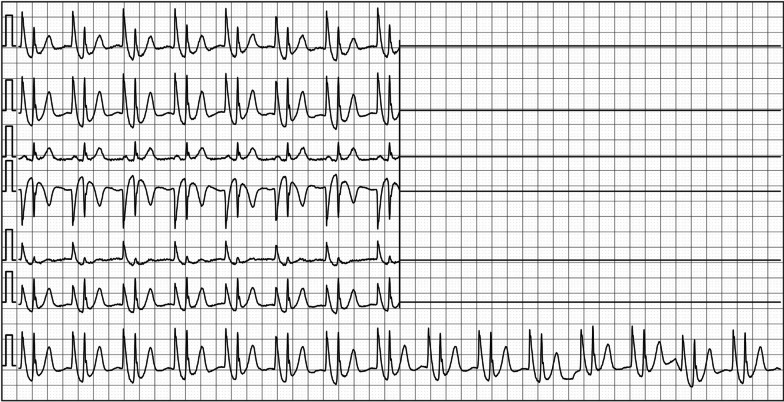
The P wave has the highest amplitude and the catheter position is appropriate

**Fig. 2:**
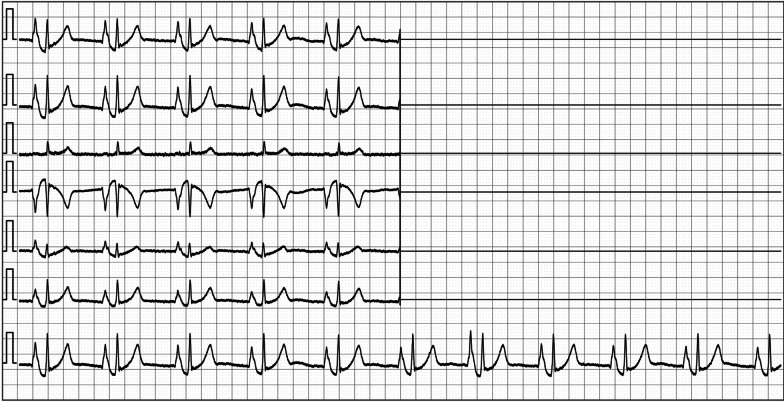
The P wave has a small negative wave and the position of the tube is appropriate

The PICC placement in ECG group was guided by intracavitary electrocardiogram, and the detailed process was as follows: (1) before operation, the responsible nurse informed the patients in detail about the function and effect of the new technology of using defibrillator or ECG monitor to guide catheterization. The target vessels were punctured, and the 10 cm arm circumference at the puncture point was measured. In the process of puncture under the guidance of B-ultrasound, a bedside vascular ultrasound machine was used. Facing the responsible nurse, and the blood vessels were selected and marked at the midpoint of the upper 1/3 of the upper arm. The patients were monitored by defibrillator or ECG monitor, the second lead ECG was adjusted, and the original data of ECG waveform were printed. (2) The length of the catheterization was jointly predicted by the responsible nurse and the chief nurse. The patient laid flat, and the abduction of the arm was 90° with the body. Segment A indicated the distance from the puncture point along the vein to the highest point of the sternoclavicular surface of the right clavicle. Segment B indicated the length of the retroflex downward to the third intercostal space. The maximum aseptic barrier was established, the towels on the treatment bed was disinfected, the ECG monitoring right arm lead was removed and the ECG signal conversion clip was connected and stored into the aseptic protective cover. (3) After puncturing the delivery tube, the length of the catheter should be measured again with the chief nurse, and the guide wire should be withdrawn to the pre-trimmed catheter scale minus 0.5 cm to trim the catheter, and (A + B + 3 + 4) cm should be used as the length of the trimmed catheter. When slowly sending the tube to the axillary vein, the patient should turn his head to the puncture side in order to avoid the catheter entering the internal jugular vein. After sending the A-segment catheter, the guide wire of the catheter should be clamped with a signal conversion clamp. After the normal ECG waveform was displayed by the defibrillator or monitor, continue to be fed through the B segment of the catheter, 1 cm each time. If there is blood return, draw out and flush the tube at any time. (4) The increased amplitude of P wave, the reaches of 30%-50% of the amplitude of QRS wave, the reaches of 50%-80% of the amplitude of QRS wave, and the waveforms of bi-directional P wave were recorded as P1, P2, P3 and P4, respectively. The lengths of the corresponding catheter B into the superior vena cava were represented by B1, B2, B3 and B4. After the relationship between the amplitude of P wave and the amplitude of QRS wave corresponding to the insertion length of segment B was confirmed by the responsible nurse and the chief nurse, the responsible nurse recorded and printed the waveform ECG (Figs. 1, 2). The catheter entered the right atrium until the P4 wave appeared, the best positioning length of the catheter was to withdraw the catheter until the P3 wave appeared, the guide wire was removed, and the catheter was flushed and fixed. (5) The X-ray film was taken, and the radiologist who had worked in the radiology department of our hospital for more than 8 years confirmed the position of the tip of the catheter. If the tube is too deep or too shallow, it should be adjusted and fixed again after returning to the ward. In the process of catheterization, if there is no P3, P4 wave or no P wave amplitude, the catheter may be ectopic, then the 20 cm of catheter should be withdrawn, and the patient should be informed that the mandible should be close to the shoulder of the tube, and the responsible nurse should push the normal saline while sending the tube to adjust.

The chest CT group was given chest CT tomography, and the CT imaging was taken in our hospital. The vertical distance from the right sternoclavicular joint to the right upper edge of the heart shadow was measured on CT, and the measured data were recorded. After the patient laid flat, the abduction arm was 90°. The responsible nurse recorded the value from the puncture point to the right sternoclavicular joint, combined with the vertical distance between the sternoclavicular joint and the right upper edge of the heart shadow measured by CT. Chest X-ray was used to locate the catheter after placement.

### Observation indexes

*Outcomes*: the tip length of the catheter in the superior vena cava, the measured length of the catheter, the actual insertion length of the catheter, the best catheterization rate, one-time placement rate of the catheter tip positioning, incidence of related complications, comfort and satisfaction were recorded and compared between the two groups.

We defined the position of the head of the catheter [7] according to the American Society of Vascular Pathway and the American Society of intravenous Infusion Nurses. The distal tip of PICC should be located in the lower 1/3 of the superior vena cava near the junction of the superior vena cava and the right atrium. From the chest X-ray, the vena cava-atrium joint is usually located in the third or fourth intercostal space, about 5 cm below the tracheobronchial angle and 3 cm to 4 cm below the Carina, so the standard ruler is used to measure the distance of the distal tip of catheter below the Carina, and the position of the catheter is accurately in the 0 cm to 4 cm below the Carina.

The best catheterization rate: t The best tube placement rate = the number of cases in the best location/the total number of cases × 100% applied by a skilled radiologist.

One-time placement rate of the tip positioning: One-time placement rate = the number of cases with one-time placement/ the total number of cases × 100%.

Incidence of related complications: the occurrence of complications such as catheter blockage, extubation, thrombus and the lower edge of T5 by chest X-ray in the two groups during treatment were recorded in detail.

Comfort [8]: the comfort of the patients in the two groups was evaluated by the general comfort questionnaire (GCQ). The scale consists of four aspects, including psychology, social culture, physiology and environment, with a total of 28 items, a score of 1–4 and a full score of 28–112. High score represents the high level of comfort in patients.

Satisfaction: using the satisfaction questionnaire of our hospital, with a total of 10 items and a full score of 0–100. The patients were evaluated with self-made satisfaction questionnaire in our hospital. The full score: 100; ≥ 85: very satisfied; 84–70: satisfied; 69–60: basically satisfied; < 60: unsatisfied. High score represents the high level of satisfaction. Satisfaction rate = (very satisfied + satisfied + basically satisfied) / total number of cases × 100%.

### Statistical analysis

SPSS 21.0 software was used to analyze the data. The measurement data were expressed by x ± s, t-test was applied, and the counting data was expressed by rate (%). Moreover, chi-square χ^2^ test was used. A P < 0.05 was considered statistical significance.

## Results

### Comparison of the tip length of the catheter in the superior vena cava, the measured length and the actual length of the catheter between the two groups

In this study, there was no significant difference in the superior vena cava length between two groups. Besides, the measured and actual lengths of catheter were measured and compared respectively. The results showed no significant difference in the measured length of catheter and the actual length of catheter placement between ECG group and chest CT group, respectively (P > 0.05), as shown in Table 2, indicating the accuracy of procedure in both groups.

**Table 2:** Comparison of the tip position of the catheter in the superior vena cava length, the catheter measured length and the actual catheter placement length between the two groups (x ± s, cm)

Group	n	Superior vena cava length	Catheter measured length	Actual catheter placement length
ECG group	218	7.48 ± 1.02	32.12 ± 3.28	32.59 ± 3.18
Chest CT group	218	7.59 ± 0.99	32.01 ± 2.98	32.31 ± 0.76
t		− 1.143	0.366	1.137
P		0.254	0.715	0.256

### Comparison of the best catheterization rate at the tip of the catheter between the two groups

The certificated radiologists confirmed the best position for tip of the catheter as the third intercostal space. The best catheterization rate at the tip of the catheter in the ECG group was significantly higher than that in the chest CT group (99.08% vs 95.87%, χ^2^ = 4.571, P < 0.05), as shown in Table 3.

**Table 3:** Comparison of the best catheterization rate at the tip of the catheter between the two groups n (%)

Group	n	Best position rate	Too shallow	Too deep
ECG group	218	99.08 (216/218)	0.46 (1/218)	0.46 (1/218)
Chest CT group	218	95.87 (209/218)	2.29 (5/218)	1.83 (4/218)
χ^2^		4.571		
P		0.033		

### Comparison of one-time placement rate of catheter tip between the two groups

The one-time placement rates of the tip of the catheter were high in both groups, but the ECG group was higher than that in the chest CT group (96.79% vs 92.20%, P < 0.05), as shown in Table 4.

**Table 4:** Comparison of one-time placement rate of catheter tip between the two groups n (%)

Group	n	One-time placement rate	Two times or more
ECG group	218	211 (96.79)	7 (3.21)
Chest CT group	218	201 (92.20)	17 (7.80)
χ^2^		4.409	
P		0.036	

### Comparison of the incidence of related complications between the two groups

The occurrence of complications such as catheter blockage, extubation, thrombus and the lower edge of T5 by chest X-ray in the two groups during treatment were recorded. There was no significant difference in the total incidence of related complications between the ECG group and the chest CT group (10.55% vs 15.60%, χ^2^ = 2.442, P > 0.05), as shown in Table 5, demonstrating both treatments were safe.

**Table 5:** Comparison of the incidence of related complications between the two groups n (%)

Group	n	Osmosis of blood	Extubation	Thrombus	Occupied space on inferior margin of chest radiography	Others	Total rate of related complications
ECG group	218	1	9	1	1	11	10.55 (23/218)
Chest CT group	218	1	11	1	3	18	15.60 (34/218)
χ^2^							2.442
P							0.118

### Comparison of comfort between the two groups

All patients received GCQ, and the total scores were calculated and compared. The higher scores they got, the more comfortable they felt. The comfort degree of ECG group was significantly higher than that of chest CT group (P < 0.05), as shown in Table 6.

**Table 6:** Comparison of patient comfort between the two groups (x ± s, points)

Group	n	Psychology	Social culture	Physiological	Environment	Total score
ECG group	218	17.09 ± 2.01	19.32 ± 2.13	19.83 ± 2.09	21.03 ± 3.09	77.27 ± 9.32
Chest CT group	218	16.67 ± 2.11	18.09 ± 1.98	18.73 ± 2.01	20.01 ± 1.76	75.26 ± 7.86
t		2.218	6.245	5.601	4.235	2.434
P		0.034	< 0.001	< 0.001	< 0.001	0.015

### Comparison of patient satisfaction between the two groups

The higher scores they got, the more satisfied they were in self-made satisfaction questionnaire. The satisfaction of ECG group was significantly higher than that of chest CT group (99.08% > 95.41%, P < 0.05), as shown in Additional file 1: Table S1.

## Discussion

Ectopic PICC catheter can lead to venous thrombosis, catheter blockage and other related complications, which has a serious impact on the prognosis of patients. When the vertical distance between the tip of the catheter and the sternoclavicular joint is close to the right upper edge of the heart shadow, the catheter is easily affected by vascular blood flow and can float freely in the vascular lumen without touching the vascular wall, which can prevent the catheter and drug solution from stimulating the vascular intima, and inserting the catheter too deep into the right atrium will lead to arrhythmia. In severe cases, it can lead to serious complications such as cardiac tamponade. Chemical phlebitis can be induced if the tube is placed too shallow. At present, X-ray examination is an effective method for positioning PICC catheters. However, X-ray fails to show the pathological changes of patients with tumor metastasis pressing superior vena cava and vascular variation, which makes it difficult to insert PICC catheter. Therefore, the use of positive and effective guidance has important clinical value as it can reduce the incidence of related complications in patients who need PICC placement, and improve the level of prognosis.

In clinical practice, the safety and effective use of PICC placement is to accurately predict the length of it. The study by Yang et al. [9] has confirmed that the correct position of the tip of PICC catheters is 44% to 99%. Copious studies are available to show that, the tip position of PICC catheters plays an important role in clinical practice [10]. According to Venkatesan et al. [11], the tip of the PICC catheter should be placed at the junction between the inferior 1/3 of the superior vena cava and the right atrium. The 2016 edition of the guidelines of the American Society of intravenous Infusion Nursing [7] specifically points out that the tip of PICC is located in the superior vena cava, and the best tip position is near the junction of the right atrium and superior vena cava. The superior vena cava is about 5–7 cm in length and is a relatively thick venous trunk, which is formed by the confluence of left and right brachiocephalic veins, located behind the right sternocostal junction, straight downward along the right side of the ascending aorta, to the lower edge of the 2 sternocostal joint into the right atrium. Therefore, in the present study, the length of superior vena cava, the length of catheter measurement and the actual length of catheter placement were analyzed under the guidance of ECG and chest CT tomography. Based on our results, the length of superior vena cava, the length of catheter measurement and the actual length of catheter placement were comparable between the two groups (P > 0.05). But the best catheterization rate at the tip of catheter in ECG group was significantly higher than that in chest CT group (P < 0.05). It was suggested that the accurate length of superior vena cava, the length of catheter measurement and the actual length of catheter placement can be obtained by ECG guidance and chest CT in the prediction of PICC placement length. In PICC placement, the best catheterization rate of catheter tip guided by IC-ECG was higher, and the best catheterization rate of chest CT was as high as 95.87%, indicating that the best catheterization rate of catheter tip of chest CT in PICC placement was second only to that of electrocardiogram guidance, and the two methods are accurate and effective.

Studies supported by Singh et al. [12] and Wu et al. [13] have confirmed that when the tip of the catheter was located in the non-central vein, the incidence of local complications such as catheter blockage, thrombosis, local phlebitis and so on increased, so the accuracy of the catheter is very important. Therefore, in this study, we compared the one-time placement rate of chest CT and intracavitary electrocardiogram in PICC placement. The results showed that the one-time placement rate of catheter tip in ECG group was significantly higher than that in chest CT group (P < 0.05). It was suggested that intracavitary electrocardiogram guidance in PICC placement can improve the one-time placement rate of catheter tip. It is worth mentioning that during the guidance of intracavitary electrocardiogram, there were 7 patients whose ECG did not show P3 wave and P4 wave, which may be that the catheter entered the azygos vein and did not succeed after many times of intubation. However, in the process of clinical operation, surgeon should avoid accidents such as blind pursuit of two-way waves and abnormal heart rate caused by excessive delivery of tubes. In clinical practice, the gold standard to determine whether PICC placement is successful or not is to take chest X-ray, and to adjust the tip position of PICC placement again. But the patients will have to receive multiple X-ray radiation. According to Anestis et al. [14], the electrocardiogram localization has high safety and accuracy in newborns, and achieved 98.8% accuracy when using ECG monitor. In the process of catheterization, the catheter can be guided to enter the superior vena cava according to the changes in the shape and amplitude of P wave, and chest X-ray is not necessary for patients with P wave characteristics. The specific position of the tip of PICC placement should be observed with the use of chest X-ray. The study by Chu et al. [15] confirmed that the accuracy of the localization method guided by intracavitary electrocardiogram was verified by TEE. The results showed that intracavitary electrocardiogram guidance could predict the tip position of the catheter more accurately than X-ray localization. The study by Wu et al. [16] has demonstrated that when the P wave was at the height of 50% of the QRS wave, the tip placement rate of the catheter was as high as 100%, with 100% specificity and sensitivity.

Based on the aggregated results of our study, the incidence of related complications in the ECG group was comparable with that in the chest CT group (P > 0.05), suggesting that the IC-ECG guidance in the PICC catheter would not increase the complications. The results may be due to the use of electrocardiogram guidance in PICC placement to obtain synchronous and real-time monitoring of the tip of the catheter to reach the best position, and to operate under aseptic technology, which can avoid secondary reverse adjustment of the catheter, reducing repeated pulling or pulling of the catheter as well as reducing the stimulation to blood vessels, resulting in a reduction in the incidence of mechanical phlebitis and related complications. Considering PICC placement is an invasive treatment operation, the psychological state of the patients tends to be poor after the disease, especially after the tumor. And the low accuracy of PICC placement can lead to reduced comfort and satisfaction of the patients, as well as increase the negative emotions, which is not conducive to the rehabilitation of the patients. In our study, the comfort and satisfaction of patients were analyzed. The results showed that the comfort and satisfaction of patients in ECG group were higher than those in chest CT group (P < 0.05), indicating that the guidance of electrocardiogram in the middle cavity of PICC tube can improve the comfort and satisfaction of patients. The outcome may attribute to the accurate actual placement length of the PICC catheter guided by intracavitary electrocardiogram, and the higher one-time placement rate. Therefore, it can relieve the tension of patients and improve their comfort and satisfaction.

The position of the catheter tip would change with the patient's body position, limbs, breathing, diaphragm and so on. Therefore, common methods in clinical observation had their limitations for severely bedridden patients. Chest CT Images had the advantages of no overlap of CT images, clear images and accurate location of lesions. Multi slice spiral CT provides a powerful vascular imaging function, which can accurately measure the vertical distance between the sternoclavicular joint and the upper right edge of the heart shadow. However, its expensive cost and extra radiation exposure would limit its application in the clinic. Doctors should choose this method according to the actual condition of patients.

However, there were some limitations in this study. First, this outcome was from a single center. It remained to be proved by a multi-central and prospective study. Besides, the sample size was a bit small. We didn’t perform subgroup analysis for age, BMI, etc. Further work should focus on these potential factors.

## Conclusion

Taken together, the accurate length of superior vena cava, the length of catheter measurement and the actual length of catheter placement could be achieved by both chest CT and ECG guidance in PICC placement. Nevertheless, intracavitary electrocardiogram elicited superior results in terms of the higher best catheterization rate and one-time placement rate, with reduced complications and high level of comfort and satisfaction, which is considered a valid approach for wide clinical application.

## Supplementary Information

**Additional file 1: Table S1.** Comparison of patient satisfaction between the two groups (%, score).

## Data Availability

The datasets generated during and/or analyzed during the present study are not publicly available, but are available from the corresponding author on reasonable request.

## Abbreviations

CT: Computed Tomography
ECG: Electrocardiogram
PICC: Peripherally inserted central catheter
