# Supplementary material for: Chest CT tomography vs. intracavitary electrocardiogram guidance in predicting the length of PICC placement
Source: BMC Surg. 2022 May 19;22:197. doi: 10.1186/s12893-022-01604-0 (PMC9118803; doi:10.1186/s12893-022-01604-0)
Supplement: Supplementary file 1 — Additional file 1: Table S1. Comparison of patient satisfaction between the two groups (%, score). [file 12893_2022_1604_MOESM1_ESM.docx]

Supplemental Table 1 Comparison of patient satisfaction between the two groups (%, score)

| Group | n | Unsatisfied | Basically satisfied | Satisfied | Very satisfied | Satisfaction rate |
| --- | --- | --- | --- | --- | --- | --- |
| ECG group | 218 | 2 | 8 | 56 | 152 | 99.08(216/218) |
| Chest CT group | 218 | 10 | 16 | 89 | 103 | 95.41(208/218) |
| t |  |  |  |  |  | 5.484 |
| P |  |  |  |  |  | 0.019 |
